# Molecular Mechanism of Exogenous GABA in Regulating Salt Tolerance in Tomato (*Solanum lycopersicum* L.)

**DOI:** 10.3390/ijms26115145

**Published:** 2025-05-27

**Authors:** Huifang Liu, Jiayi Xing, Qiang Wang, Yanan Chang, Hongmei Zhuang, Hongwei Han, Rong Zhou, Hao Wang, Huiying Liu

**Affiliations:** 1 Laboratory of Genome Research and Genetic Improvement of Xinjiang Characteristic Fruits and Vegetables, Institute of Fruits and Vegetables, Vegetable Engineering Technology Research Center, Xinjiang Academy of Agricultural Sciences, Urumqi 830002, China; huifangliu@xaas.ac.cn (H.L.); jiayix2021@xaas.ac.cn (J.X.); yananchang1992@xaas.ac.cn (Y.C.); zhuanghongmei@xaas.ac.cn (H.Z.); hhwei2010@sohu.com (H.H.); wanghao@xaas.ac.cn (H.W.); 2 Sanya Research Institute, Nanjing Agricultural University, Nanjing 210095, China; zhour@njau.edu.cn; 3 Department of Food Science, Aarhus University, 8200 Aarhus, Denmark; 4 Key Laboratory of Special Fruits and Vegetables Cultivation Physiology and Germplasm Resources Utilization of Xinjiang Production and Construction Crops, Department of Horticulture, Agricultural College, Shihezi University, Shihezi 832000, China; hyliuok@aliyun.com

**Keywords:** GABA, tomato, salt stress, tomato, plant signal transduction, carbon metabolism

## Abstract

To explore the mechanism by which γ-aminobutyric acid (GABA) regulates the response of different salt-sensitive tomato seedlings under salt stress conditions, we used the previously selected salt-sensitive tomato ‘M82’ and the salt-tolerant introgression line ‘IL-7-5-5’. The following three treatments were set up: (1) a normal nutrient solution concentration as the control, (2) a nutrient solution with 200 mmol·L^−1^ NaCl, and (3) a nutrient solution with 200 mmol·L^−1^ NaCl and 35 mmol·L^−1^ GABA. The concentration of the reactive oxygen species metabolism-related compounds and antioxidant enzyme activity in the leaves of tomato seedlings subjected to the different treatments were measured, and transcriptome and metabolome analyses were conducted. After adding GABA, the SOD, POD, and APX activity in the leaves of the ‘M82’ seedlings significantly increased, while the GR activity significantly decreased. In the ‘IL-7-5-5’ seedlings, the CAT, APX, and GR activity significantly increased. The combined results from the transcriptome and metabolome analysis in leaves indicated that in ‘M82’ seedlings, 52 metabolic pathways were enriched, which included plant signal transduction pathways, phenylpropanoid biosynthesis pathways, and amino sugar and nucleotide sugar metabolism pathways. In the salt-tolerant ‘IL-7-5-5′ seedling leaves, 59 metabolic pathways were enriched, which included plant signal transduction pathways, amino acid biosynthesis pathways, and carbon metabolism pathways. A further analysis revealed that both varieties had a higher number of differentially enriched genes and differential metabolites belonging to the plant hormone signal transduction and amino acid biosynthesis pathways, indicating that GABA enhances the salt tolerance of tomato seedlings by regulating these two mechanisms.

## 1. Introduction

The tomato (*Solanum lycopersicum* L.) is an annual vegetable crop in the Solanaceae family, which is moderately sensitive to salt stress throughout its development. Extensive research has shown that applying exogenous substances, such as γ-aminobutyric acid (GABA), potassium humate (HA-K), salicylic acid (SA), and nitric oxide (NO), can enhance the salt tolerance of crops [1,2,3,4,5]. Among them, GABA, as a four-carbon non-protein amino acid, is commonly present in plants and is an important plant signaling molecule. It acts as a nutritional substance or metabolic regulator, playing a regulatory role in the growth and development of roots, stems, leaves, flowers, fruits, seeds, and other organs under various environmental conditions [6,7]. A wealth of research has indicated that GABA plays a role in signal transduction in plants responding to abiotic stress by regulating the primary metabolism, gene expression, and ion homeostasis and inducing the antioxidant defense [8,9]. Hany et al. (2021) found that exogenous GABA alleviates the growth inhibition caused by drought and increases the yield and quality of cowpeas by maintaining the cell membrane integrity, alleviating osmotic damage, activating the antioxidant defense, and increasing the accumulation of nutrients [10]. Another study conducted on tea plants (*Camellia sinensis* (L.) *O. Kuntze*) showed that exogenous GABA induced an increase in GABA levels, altered the levels of stress response compounds, and induced interactions among photosynthesis, amino acid biosynthesis, and carbon–nitrogen metabolism, thereby enhancing cold tolerance in the tea plants [11]. These studies confirmed that exogenous GABA can effectively improve plants’ stress tolerance by regulating gene expression and metabolic products, which enhanced our understanding of GABA’s role in stress adaptation.

Our previous study revealed that the high expression of three key protein-coding genes in tomato leaves involved in GABA biosynthesis regulated a series of responses in plants responding to salt stress [12]. This study further investigated the physiological and biochemical changes in tomato seedlings under salt stress conditions with exogenous GABA using a combination of chemical analysis, bioinformatics methods, transcriptomics, and metabolomics. Differentially expressed genes and metabolites in tomato seedlings under salt stress conditions with exogenous GABA were identified. This research significantly advances our understanding of crop salt tolerance mechanisms and contributes to the development of salt-tolerant tomato germplasms.

## 2. Results

### 2.1. GABA Regulation of MDA, H_2_O_2_, and O_2_^−^ Content in Tomato Seedling Leaves Under Salt Stress Conditions

**Figure 1:**
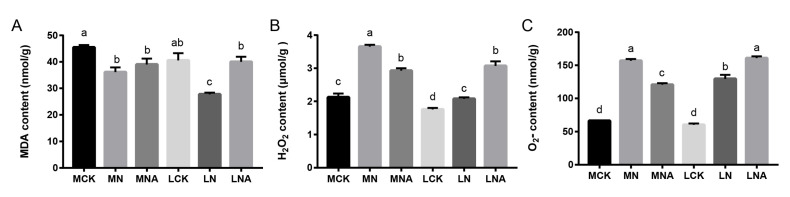
The MDA, H_2_O_2_, and O_2_^−^ content in the leaves of tomato seedlings under salt stress. ((**A**): MDA content; (**B**): H_2_O_2_ content; (**C**): O_2_^−^ content). Note: Different lowercase letters indicate significant differences between treatments (*p* < 0.05). The following is the same.

The changes in the MDA (Malondialdehyde) content in the tomato seedling leaves of both varieties followed a consistent trend, with a significant decrease under NaCl stress conditions, whereas the exogenous GABA application increased the MDA content, with the IL7-5-5 cultivar showing a significant increase (Figure 1A, *p*-value is in Table 1). The changes in the H_2_O_2_ (Hydrogen Peroxide) and O_2_^−^ (Superoxide) content were also consistent, showing a significant increase in both M82 and IL7-5-5 seedlings under NaCl stress conditions as compared with the control (Figure 1B,C). This indicated that NaCl stress led to the accumulation of H_2_O_2_ and O_2_^−^ in the leaves of tomato seedlings (Figure 1B,C, *p*-value is in Table 1). After adding GABA, the H_2_O_2_ and O_2_^−^ content in the leaves of the M82 tomato seedlings significantly decreased but to levels that were still significantly higher than in the control, while the H_2_O_2_ and O_2_^−^ content in the leaves of the IL7-5-5 seedlings significantly increased (Figure 1B,C). This suggests that adding GABA can regulate the H_2_O_2_ and O_2_^−^ content in the leaves of tomato seedlings under NaCl stress conditions, but the response mechanisms in the two varieties are different.

The tomato varieties used were the salt-tolerant introgression line IL7-5-5 and the salt-sensitive cultivated tomato M82. The experiment included three treatments: (1) the cultivation in a 1× Hoagland nutrient solution as the control (MCK, LCK); (2) a nutrient solution + 200 mmol·L^−1^ NaCl (MN, LN); and (3) a nutrient solution + 200 mmol·L^−1^ NaCl + 35 mmol·L^−1^ GABA (MNA, LNA). The following is the same.

### 2.2. GABA Regulates Antioxidant Oxidase Activity in Tomato Seedling Leaves Under Salt Stress

**Figure 2:**
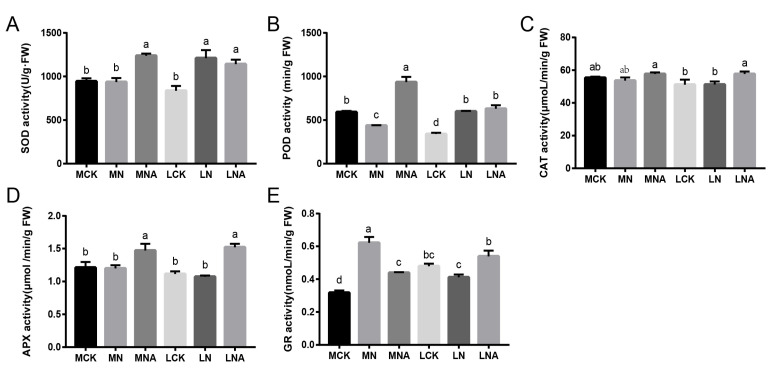
The antioxidant activity in the leaves of tomato seedlings under salt stress. ((**A**): SOD activity; (**B**): SOD activity; (**C**): CAT activity; (**D**): APX activity; (**E**): GR activity).

As shown in Figure 2, compared with the control, the POD (Peroxidase) activity in the leaves of the M82 seedlings was significantly lower and the GR (Glutathione Reductase) activity was significantly higher under the NaCl stress (Figure 2B, *p*-value is in Table 2). The SOD (Superoxide Dismutase) and POD activity in the leaves of the IL7-5-5 seedlings was significantly higher. These results indicate that the two tomato varieties respond to NaCl stress by adjusting the activity levels of SOD, POD, GR, and other enzymes (Figure 2A,B,E, *p*-value is in Table 2). After adding GABA, the SOD, POD, and APX activity in the leaves of the M82 seedlings was significantly higher, while the GR activity was significantly lower. The CAT (Catalase), APX (Ascorbate Peroxidase), and GR activity (Figure 2C,D, *p*-value is in Table 2) in the leaves of the IL7-5-5 seedlings was significantly higher. These results indicate that GABA can improve the active oxygen scavenging ability of tomato seedling leaves by increasing the activity levels of antioxidant enzymes.

### 2.3. Metabolite Accumulation

**Figure 3:**
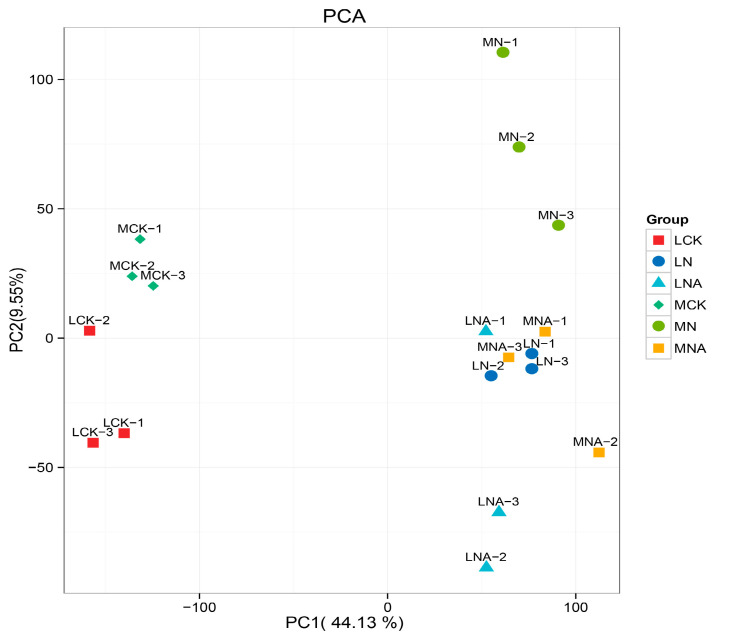
The principal component analysis. Note: Each point represents a sample, and samples in the same group are represented with the same color. The numbers associated with each data point represent the biological replicates.

The targeted metabolome analysis on 18 samples detected and quantified 944 metabolites. They included 124 amino acids; 121 terpenes; 101 organic acids; 96 sugars and alcohols; 87 alkaloids; 82 lipids; 63 flavonoids; 43 polyphenols; 33 nucleotides; 31 vitamins; 31 ketones, aldehydes, and acids; 23 steroids; 20 phenylpropanes; 8 quinones; 8 coumarins; 7 lignans; and 2 oxanthrones. These results indicate that the metabolite composition of the two varieties of tomato seedlings was significantly altered under the NaCl stress, and it also changed after GABA was added. The replicates also clustered consistently by sample, except for MNA-2, indicating that the sample-to-sample variation and treatment effects were greater than the variability introduced during the sample handling process. The PCA results shown in Figure 3 also illustrate this point.

### 2.4. Analysis of Differentially Accumulated Metabolites

An orthogonal partial least squares discriminant analysis (OPLS-DA) was performed on all low-molecular-weight metabolites in the samples to screen for differential variables while minimizing the uncorrelated variation. According to the OPLS-DA results, the variable importance projection (VIP) value > 1 combined with fold changes ≥2 and ≤0.5 were used to further screen for differentially accumulated metabolites. The number of differential metabolites in the leaves of M82 seedlings can be seen in Table 3. 

### 2.5. Classification and Annotation of Differential Metabolites

**Figure 4:**
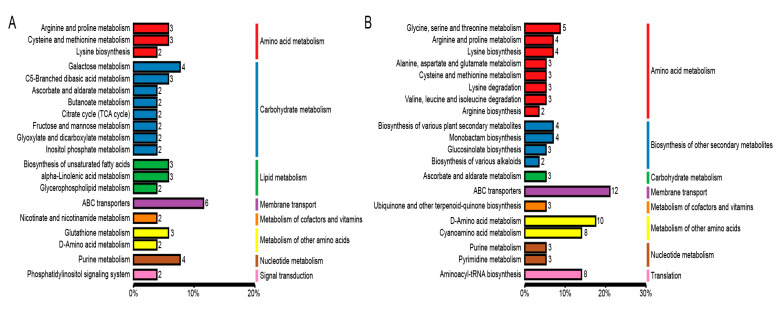
The KEGG database classification summary including (**A**) MN-MNA and (**B**) LN-LNA. The entries in the figure represent the level classification notes of the KEGG pathway, corresponding to KO pathway Level 2 and KO Pathway Level 3. The length of the column represents the number of metabolites annotated by the pathway.

Metabolites interact inside organisms to form different pathways. The metabolites detected in the tomato seedlings were annotated using the KEGG database, and the annotation results were classified according to the different pathway types in the KEGG. The results showed that the differential metabolites belonged to 95 pathways, among which the differential metabolites of MN vs. MNA belonged to 53 pathways (Figure 4A), and those of LN vs. LNA belonged to 68 pathways (Figure 4B). This indicated that the two tomato varieties respond to GABA by regulating the metabolic activity of different pathways.

In order to deepen our understanding of the role of differential metabolites in the tomato seedling leaves from the two varieties, a KEGG pathway enrichment analysis was performed on the differential metabolites. The metabolites belonging to the ABC transporters, galactose metabolism, and purine metabolism were enriched among the differential metabolites in MN vs. MNA. In LN vs. LNA, those belonging to the ABC transporters, the D-Amino acid metabolism, and the Cyanoamino acid metabolism were enriched.

### 2.6. Quality Assessment of RNA-Seq Data from Tomato Seedling Leaves Under Different Treatment Conditions

A total of 117.26 Gb of clean data were obtained from the 18 samples from the two tomato varieties (Table 4). Each sample contained 5.71 Gb, and the Q30 read quality of all the samples was greater than 96%. This indicates that the sequencing quality of this experiment is high, and the data can be analyzed further. The Q30 (%) between the clean reads and tomato reference genome ranged from 96.71% to 98.28%.

### 2.7. The Comparative Analysis with the Reference Genome

The cleaned reads were aligned to the reference genome using HISAT2 to determine their genomic location and the characteristics of the sequenced samples. The very high percentage of reads mapping to unique locations in the reference genome indicated that the data obtained for this experiment are credible and can be analyzed further (Table 5).

### 2.8. KEGG Enrichment Analysis of Differentially Expressed Genes

**Figure 5:**
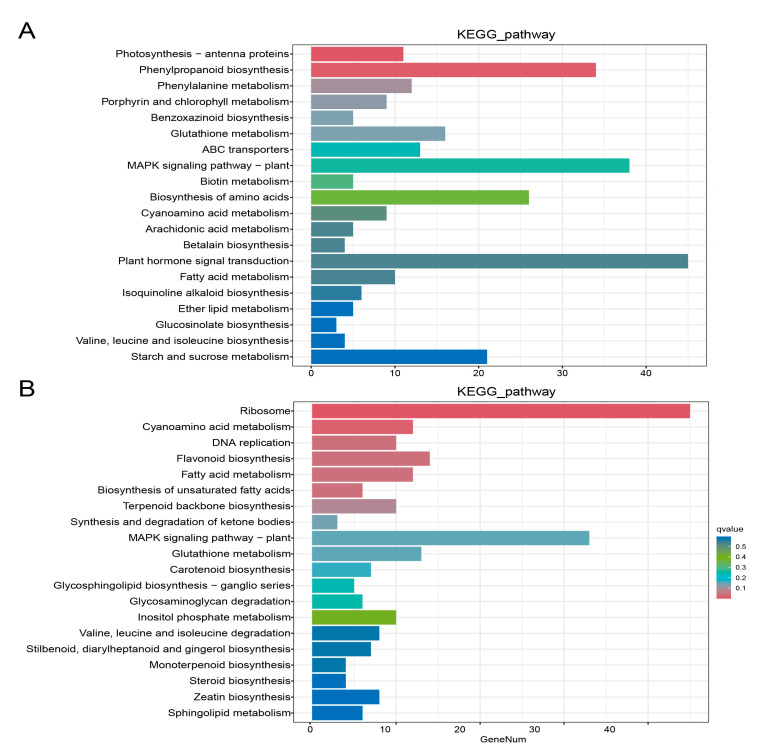
KEGG enrichment analysis of different genes in (**A**) MN-MNA and (**B**) LN-LNA.

In order to analyze the role of differentially expressed genes in the tomato seedling leaves belonging to the two varieties, a KEGG pathway enrichment analysis was performed on the differentially expressed genes. Figure 5 lists the top 20 pathways with the lowest significant q values. The genes belonging to the plant hormone signal transduction and the MAPK signaling pathway—plants, phenylpropyl biosynthesis, amino acid biosynthesis, and starch and sucrose metabolism—were enriched among the differentially expressed genes between MN and MNA. Those belonging to the ribosomes and MAPK signaling pathways—plants, flavonoid biosynthesis, glutathione metabolism, Cyanoamino acid metabolism, and fatty acid metabolism—were enriched among the differentially expressed genes between LN and LNA.

### 2.9. Association Analysis of Differential Metabolites and Differentially Expressed Genes

The KEGG metabolic pathways with significant enrichments of differentially expressed genes and differential metabolites were mapped to each other and analyzed to identify overlapping pathways. The results showed that 52 and 59 identical metabolic pathways were enriched between the MN and MNA treatment groups and the LN and LNA treatment groups, respectively. In order to determine the relationship between the genes and metabolites associated with GABA-mediated salt stress, those identified in this study were counted, and the top 10 KEGG pathways with the highest number of genes and metabolites were visualized, which included the plant signal transduction pathways (plant hormone signal transduction), MN vs. MNA treatment group differences in genes (protein phosphatase 2C, small auxin protein, etc.), and one difference metabolite (corn). Between the LN and LNA treatment groups, twenty-eight differentially expressed genes (e.g., protein phosphatase 2C and PR1 protein precursor) and one differential metabolite (salicylic acid) was enriched. In the biosynthesis of the amino acids pathway, seventeen differentially expressed genes (e.g., asparagine synthetase and threonine dehydrase) and two differential metabolites (adipic acid and proline) were enriched between the MN and MNA treatment groups. Between the LN and LNA groups, 16 differentially expressed genes (e.g., threonine dehydrase and enolase) and 10 differential metabolites (aspartic acid and shikimic acid) were enriched. In the carbon metabolism pathway, twenty-eight differentially expressed genes (e.g., auxin response protein 15 and ethylene response transcription factor) and one differential metabolite (malic acid) was enriched between the MN and MNA treatment groups. Between the LN and LNA treatment groups, sixteen differentially expressed genes (e.g., formate dehydrogenase and threonine dehydrase) and three differential metabolites (gluconic acid, aspartate, and serine) were enriched.

### 2.10. qRT-PCR Verification Analysis

**Figure 6:**
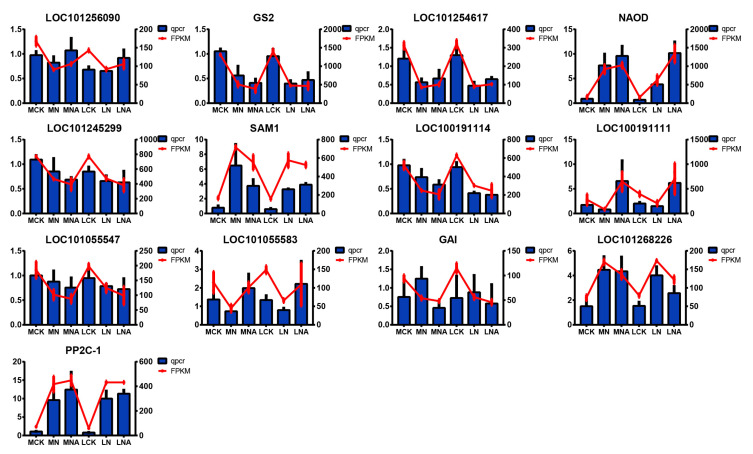
qRT-PCR analysis of selected key genes. LOC101256090: serine hydroxymethyltransferase 4, GS2: glutamine synthetase, LOC101055583: small auxin-up protein 58, LOC101268226: transcription factor UNE10, LOC101254617: cysteine synthase, chloroplastic/chromoplastic isoform X1, NAOD: N2-acetylornithine deacetylase, LOC101055547: IAA14, LOC101245299: transketolase, chloroplastic, GAI: DELLA protein GAI, SAM1: S-adenosylmethionine synthase 1, LOC100191111: PR1 protein precursor, LOC100191114: jasmonate ZIM-domain protein 3, and PP2C-1: protein phosphatase 2C ABI2 homolog.

Alongside the transcriptome gene expression analysis, 13 genes were also selected for the qRT-PCR to confirm their response to the exogenous GABA amendment under salt stress conditions (Figure 6), including (Following the order from left to right and from top to bottom) LOC101256090: serine hydroxymethyltransferase 4, GS2: glutamine synthetase, LOC101055583: small auxin-up protein 58, LOC101268226: transcription factor UNE10, LOC101254617: cysteine synthase, chloroplastic/chromoplastic isoform X1, NAOD: N2-acetylornithine deacetylase, LOC101055547: IAA14, LOC101245299: transketolase, chloroplastic, GAI: DELLA protein GAI, SAM1: S-adenosylmethionine synthase 1, LOC100191111: PR1 protein precursor, LOC100191114: jasmonate ZIM-domain protein 3, and PP2C-1: protein phosphatase 2C ABI2 homolog. The qRT-PCR results for all the genes were consistent with the transcriptome results, indicating that the RNA-seq data in this experiment were credible. Among them, after GABA was added, the expression levels of LOC101256090, NAOD, LOC100191111, and LOC101055583 PP2C-1 were up-regulated. Notably, LOC100191111 was significantly up-regulated by 3.8 times, which makes it a potential candidate gene.

## 3. Discussion

### 3.1. GABA Enhances Antioxidant Enzyme Activity in Tomato Seedlings

GABA is a recently discovered amino acid with many biological functions. It has been shown to stimulate plant growth and development and mediate adaptive responses to biotic and abiotic sources of stress [13]. Studies have confirmed that a treatment with 5 mM of GABA can reduce the levels of Superoxide anions and Hydrogen Peroxide in strawberries under salt stress conditions, while simultaneously enhancing the antioxidant capacity of the strawberries [14]. In this study, exogenous GABA was applied to tomato seedlings to study the response mechanism of tolerant tomato varieties. The results showed that the H_2_O_2_ and O_2_^−^ content in the leaves of M82 seedlings was significantly lower (Figure 1B,C), while the SOD, POD, and APX activity was significantly higher (Figure 2A,B,D) and that of GR was significantly lower after GABA was added (Figure 2E). The MDA, H_2_O_2_, and O_2_^−^ content in the leaves of IL7-5-5 seedlings was significantly higher (Figure 1A–C), as was the CAT, APX, and GR activity (Figure 2C–E). Our study indicated that GABA could improve the active oxygen scavenging ability of tomato seedlings by increasing the activity of antioxidant enzymes, and the response mechanisms of the two tomato varieties were different. These results are consistent with those findings by Ahmad S. in tomatoes and SHU C. in rice [15,16].

### 3.2. Transcription Factors Play a Key Role in Plant Salt Stress Responses

Li et al. showed that transcription factors can improve plants’ stress resistance under high salt stress [17]. Currently, the WRKY, NAC, MYB, ZIP, AP2/ERF, and HSF families are mainly known to be involved in the salt stress response [18,19,20]. A large number of studies have found that genes, such as CabZIP25 in peppers [21], GmbZIP2 in soybeans [22], and AhbZIP in peanuts [23], are induced by salt stress, and their expression levels become significantly different when exposed to high salt (150 mmol/L NaCl) treatments. Studies have also confirmed that the UNE and TGA transcription factor family members are present in tobacco (*Nicotiana tabacum* L., NtbHLH123), wheat (*Triticum aestivum* L., TabHLH1), soybeans (*Glycine max* L., NPR1-TGA2), and Arabidopsis (*Arabidopsis thaliana*, AtbHLH18, AtbHLH34, and AtbHLH115) [24,25,26,27,28]. These factors can regulate the expression of a series of stress-related genes to improve the salt tolerance of plants. In our experiment, MYB, bHLH, AP2, C2H2, NAC, WRKY, LAPA, and other transcription factors were involved in regulating the salt tolerance in tomatoes. Compared with the control seedlings, the MYB and LAPA1 expression in the salt-sensitive and salt-tolerant varieties were up-regulated. LAPA1 is a candidate gene that regulates the tomato’s salt tolerance by adding GABA under salt stress, and the MYB12 transcription factor plays an important role in the tomato’s adaptation to salt stress.

### 3.3. Combined Transcriptomics and Metabolomics Reveal GABA’s Role in Salt Stress Adaptation

Transcriptomics and metabolomics data were combined to explore the molecular mechanism of GABA regulation to induce salt tolerance in tomatoes. GABA has been reported to have many roles during the plant’s response to abiotic stress [29,30]. We performed a KEGG annotation using both metabolome (Figure 4) and transcriptome (Figure 5) data and then conducted the annotation through the integration of the two omics analyses (Table 6 and Table 7). Adding GABA regulated the salt tolerance in both salt-sensitive and salt-tolerant tomatoes. The KEGG pathway enrichment analysis of differential genes and metabolites in MNA and LNA showed that M82 was related to plant hormone signal transduction, including the auxin response genes GH3-8, GH3-10, and small auxin-up protein 58. The small auxin-up protein 58 gene and other genes have been validated in the qRT-PCR data (Figure 6). The differentially expressed gene Auxin-Responsive protein SAUR32 in the plant hormone signal transduction pathway in IL7-5-5 indicated that adding GABA could counteract salt stress by regulating various genes in the auxin signaling pathway in both tomato varieties.

Amino acids in plants are indispensable osmotic substances, and enhancing their synthesis promotes osmotic regulation and the maintenance of cell membrane homeostasis in plants under stress conditions. Secondly, they are important antioxidants in plants, effectively removing excess free radicals and reducing salt stress-induced damage to plants [31]. These two functions complement each other and jointly contribute to improving the resilience of plants under salt stress. In pepper seedlings, exogenous GABA can improve the plants’ tolerance to salt stress by regulating the concentrations of proline and other substances in their leaves. Metabolizing proline and arginine also plays an important role when coping with salt- and alkalinity-induced stress [32]. In our experiment, the amino acid biosynthesis pathway showed a significant up-regulation in the expression of nine amino acids and metabolic enzymes in the M82 seedlings. These enzymes included aspartic acid synthetase, alanine aminotransferase 2, aspartate aminotransferase, cytoplasm arginase 1, cysteine synthase, threonine dehydrase 2, chloroplast threonine deaminase 1 precursor, phospho-2-deoxy-3-deoxyheptanoate aldolase 1 (chloroplast), and branch chain amino acid aminotransferase 2 (chloroplast). Among them, cysteine synthase is a key enzyme in the L-cysteine biosynthesis pathway in bacteria, plants, and protozoa. It plays a crucial role in essential biological processes, such as the sulfur assimilation metabolic pathway, the regulation of the REDOX balance, and the response to environmental stress. Other enzymes, such as aspartic acid synthase, regulate the osmotic pressure in tomato cells, maintain osmotic balance, and resist salt stress by influencing the synthesis and degradation of amino acids. Significant changes were observed in the expression levels of eight amino acid synthases and metabolic enzymes in IL7-5-5 seedlings. These included branched amino acid aminotransferase 2, arginase 2, asparagine synthase, 3-phosphoshikimic acid 1-carboxyvinyltransferase (chloroplast), bifunctional L-3-cyanoalanine synthase/cysteine synthase 1 (mitochondria), and tryptophan synthase β-chain 1. Among them, the branched-chain amino acid transaminase is involved in the metabolism of branched-chain amino acids (leucine, isoleucine, and valine). It may act as an osmoregulatory substance in plants responding to salt stress and help maintain the intracellular osmotic balance. This is consistent with the research conclusions of Sun Y. (2024) [33] and Wu K. (2024) [34]. Asparagine synthetase (AS) is a kind of aminotransferase that is commonly found in organisms. It uses ammonia and/or aspartic acid as a substrate to catalyze the biosynthesis of asparagine and plays an important role in the plant nitrogen metabolism and protein synthesis, which, in turn, directly affects the growth and development of plants.

ABC transporters are membrane proteins that transport various types of substances across membranes using active or passive transport [35]. They help tolerate biological and abiotic sources of stress and maintain the cell’s osmotic homeostasis, signal transduction, and lipid homeostasis [36]. In this experiment, the KEGG pathways of the two tomato varieties were examined, and the ABC transporters pathway activity was shown to be significantly different in the two tomato varieties. Between the MN and MNA treatment groups, the expression levels of the genes coding ABCB9, ABCC5, ABCG3, ABCG22, and other transporters changed significantly, and the metabolites raffinosaccharide, sulfate, and adenine (hemisulfate) were significantly up-regulated. Studies have shown that most of the identified ABCB members primarily participate in the transport and regulation of hormones (auxin), and some members are functionally redundant and can also transport auxin [37,38]. The currently identified functions of ABCC members include the transport of metabolites (e.g., anthocyanins, phytic acid, and folic acid) [39,40] and detoxification [41,42]. ABCG is the largest subfamily among the ABC transporters. At present, there are 70 known ABCG transporters in tomatoes [43] that are involved in important biological functions and can improve the plants’ resilience against abiotic sources of stress, like heavy metals [44]. In this experiment, significant changes in the expression levels of transporter genes were detected in all three subfamilies in the M82 seedlings, suggesting that GABA regulates the transport efficiency of this pathway in M82 seedlings to maintain the cell osmotic balance and actively respond to salt stress. In M82 seedlings, the up-regulation of ABCB9 is synchronized with the expression changes in the auxin-responsive gene GH3-8. Auxin-related genes, such as SAUR6, SAUR14, and SAUR16, have been proven to regulate the activity of POD or CAT, thereby affecting the cellular antioxidant capacity [45]. This suggests that GABA may indirectly regulate the antioxidant enzyme system through ABC transporters and auxin.

There were significant differences in the levels of the ABCG22 transporter gene and 18 different metabolites between the LN and LNA treatment groups, including mannitol, sorbitol, glutamine, sulfate, and L-lysine hydrochloride, which participate in various biochemical processes via different pathways. Among them, mannitol and sorbitol are osmoregulatory substances that help plants maintain an intracellular water balance under salt stress, reduce water loss by reducing the intracellular osmotic pressure, and, thus, improve salt tolerance in plants. Glutamine is an important form of nitrogen storage and transport in plants. Sulfate is a source of sulfur in plants, and its metabolism is essential in antioxidant defense and protein synthesis in plants. When studying the effects of salt on sesame, Zhang et al. found that the differentially expressed genes and differential metabolites were highly correlated with the amino acid metabolism. Compared with the salt-sensitive sesame, the accumulation of various amino acids in the salt-tolerant sesame was greater, and the expression levels of multiple genes related to amino acid synthesis were higher than those in the salt-sensitive sesame. Therefore, this is likely to be the key mechanism behind salt tolerance in sesame [46]. After adding GABA, mannitol and sorbitol significantly accumulated in IL7-5-5 seedlings, and the expression of ABCG3 and ABCG22 was up-regulated. This was consistent with the greater increase in the SOD activity (Figure 2A). This correlation suggests that GABA may enhance the oxidative stress defense capability of IL7-5-5 tomatoes by up-regulating the expression of ABCG3/ABCG22, thereby promoting the transmembrane transport of mannitol and sorbitol. The accumulation of polyols not only maintains the cellular osmotic balance but also may synergistically alleviate the oxidative damage induced by salt stress by directly scavenging hydroxyl radicals and indirectly supporting SOD activity [47,48].

Carbon metabolism is an extremely complex process involving multiple biochemical steps. It is essential for the synthesis, decomposition, and conversion of carbohydrate compounds in plants. It not only affects the growth and development process of plants but also determines the ability of plants to cope with external sources of stress [49]. In this study, the differentially expressed genes and metabolites that belonged to the carbon metabolism pathway in both tomato varieties were heavily enriched. Among them, those in the M82 seedlings included fructose diphosphate aldolase, threonine dehydrase 2, enolase, cisaconite hydratase, formate dehydrogenase, succinate dehydrogenase, and malic acid. In the IL7-5-5 seedlings, the differentially expressed genes and metabolites included glycine cracking system H protein, threonine dehydrase 2, enolase, formate dehydrogenase, D-gluconic acid, aspartate, and serine. Accumulated carbohydrates can not only effectively alleviate the impact of osmotic stress on plants but also act as an important source of energy. Bao et al. also found that sugar accumulation played a key role in maintaining the cell’s osmotic balance and cell membrane structure’s stability at low temperatures. These results indicate that regulating the carbon metabolism may be one of the main mechanisms of the GABA-induced positive response of tomato seedlings to salt stress and the different response mechanisms observed in tomato seedlings with different degrees of salt tolerance.

## 4. Materials and Methods

### 4.1. Growth Conditions and Treatments

This experiment was conducted at the vegetable experimental research facility of the Institute of Horticultural Crops, Xinjiang Academy of Agricultural Sciences, China in 2023. The tomato varieties used were the salt-tolerant introgression line IL7-5-5 and the salt-sensitive cultivated tomato M82, which were previously selected. The seeds were seeded in peat, perlite, and vermiculite (volume ratio 1:1:1) substrates, and seedlings were raised in a conventional greenhouse. The roots were cleaned when the seedlings grew to the two-true-leaf stage, then transplanted the plant into 1/2 concentration Hoagland standard nutrient solution was used for hydroponic culture [50]. They were cultivated in this solution until they reached the four-true-leaf stage, after which they were switched to 1× Hoagland nutrient solution. Different treatments were applied three days later. The experiment included three treatments: (1) cultivation in 1× Hoagland nutrient solution as the control (MCKm, LCK); (2) nutrient solution + 200 mmol·L^−1^ NaCl (MN, LN); and (3) nutrient solution + 200 mmol·L^−1^ NaCl + 35 mmol·L^−1^ GABA (MNA, LNA). Each treatment was conducted with three biological replicates and random arrangement of the plants during the experiment. The indoor environmental conditions were as follows: ambient temperature of 25–35 °C during the day and 15–20 °C at night, air humidity at 40%, and a photoperiod of 11 h of light/13 h of darkness. To prevent salt shocking the plants, NaCl was added to the nutrient solution in two separate additions over two days at 10 a.m. to achieve a final treatment concentration of 200 mmol·L^−1^. This salt concentration was selected as the half-lethal concentration based on preliminary experiments. Sampling was conducted on the third day of treatment. Each treatment had three replicates, and leaves from the same part of each plant were selected for testing. The leaves were flash frozen in liquid nitrogen and then stored at −80 °C for later use.

### 4.2. Measurement of Physiological Indicators in Tomato Seedling Leaves

The MDA (Malondialdehyde) content, H_2_O_2_ (Hydrogen Peroxide) content, and O_2_^−^ (Superoxide) content and antioxidant enzyme activities of CAT (Catalase), SOD (Superoxide Dismutase), POD (Peroxidase), APX (Ascorbate Peroxidase), and GR (Glutathione Reductase) were determined by using the kit of Suzhou Keming Biotechnology Co. (Suzhou, China). The operation procedure referred to the instruction manual of the kit. Kit was purchased from Suzhou Keming Biotechnology Co., Ltd. (Suzhou, China).

The indicators including MDA content, H_2_O_2_ content, and O_2_^−^ content and the antioxidant enzyme activity of CAT, SOD, POD, APX, and GR were measured.

### 4.3. Differential Gene Expression Analysis in Tomato Seedling Leaves

The total RNA was extracted using an extraction kit (Tiangen DP411, Beijing, China). The concentration of the extracted nucleic acids was measured using a Nanodrop 2000 (Thermo Fisher Scientific, Wilmington, DE, USA), and their integrity was assessed using LabChip GX (Waltham, MA, USA). The samples that passed quality control were used to construct cDNA libraries with the VAHTS Universal V6 RNA-seq Library Prep Kit for Illumina, followed by paired-end sequencing on the Illumina NovaSeq 6000 platform (Agilent Technologies, Santa Clara, CA, USA). The HISAT2 software (v2.2.1) was used to align the filtered clean sequencing data to the tomato reference genome (NCBI, GCF_000188115.2) and identify and quantity differentially expressed genes. DESeq2 was used for differential expression analysis. Gene function annotation analysis was conducted using the GO (Gene Ontology) and KEGG (Kyoto Encyclopedia of Genes and Genomes) databases to identify the metabolic pathways in which the differentially expressed genes are involved.

DESeq2 was used to analyze the differential expression between sample groups. After the differential expression analysis, it was also necessary to use the Benjamini–Hochberg method to perform corrections for the multiple hypothesis testing and calculate the false discovery rate (FDR). The screening conditions for differential genes were fold change ≥1.5 and FDR < 0.05. The results were counted to obtain the number of reads of each gene. Then, the gene numbers were combined to obtain the total number of differential genes, up-regulated genes, and down-regulated genes in each group.

### 4.4. Differential Metabolite Analysis in Tomato Seedling Leaves

Three biological replicate samples were sent to a biotechnology company (Beijing Biomaker Biological Technology Co., Beijing, China) for qualitative and quantitative analysis of the metabolites using broad-targeted metabolic technology (LC-MS/MS). The leaf samples stored at −80 °C were ground in liquid nitrogen and then extracted in extraction solution. The metabolite samples were analyzed using a UPLC-MS system (Waters, Milford, Milford, MA, USA) to detect metabolites, and the metabolites were identified based on secondary mass spectrometry information. Principal component analysis (PCA) and correlation analysis were performed on the samples to assess sample reproducibility and variability. Orthogonal partial least squares discriminant analysis (OPLS-DA) was used to analyze and screen significantly differential metabolites. The KEGG database was used to identify the specific biological pathways in which the differential metabolites are involved.

### 4.5. Integrated Transcriptome–Metabolome Analysis of Tomato Seedling Leaves

After combining the TPM (Transcripts Per Million) expression levels of the differentially expressed genes and differential metabolites in each sample, KEGG pathway annotation was performed on those genes and metabolites that changed significantly. The genes and metabolites that changed significantly within the same biological processes were then identified to determine the metabolic pathways involved.

### 4.6. qRT-PCR Validation of Transcriptome Sequencing Data

Total RNA was extracted from samples at specific time points using a commercial kit (Tiangen DP411, Beijing, China) following the manufacturer’s instructions. The RNA was reverse transcribed using a FastKing RT Kit (Tiangen, Beijing, China) according to the method of manufacturer’s instructions. RT-qPCR analysis was conducted using Super Real Premix Plus (SYBR Green) (Tiangen, Beijing, China). RT-qPCR analysis was performed on LOC101256090, GS, LOC101254617, NAOD, LOC101245299, SAM1, LOC100191111, LOC101055547, LOC101055583, GAI, LOC101268226, and PP2C-1. The primers used are listed in Table S1. Relative expression for each of the analyzed genes was determined using the 2^−ΔΔCt^ method. An Actin gene was used for normalization.

### 4.7. Data Processing and Analysis

Data analysis was conducted using the SPSS 17.0 statistical software. Tukey (*p* < 0.05) was performed to compare the different treatments. All experiments were replicated three times, and the results of the experiments are expressed as the mean ± standard deviation.

## 5. Conclusions

In the salt-sensitive tomato variety (M82), 52 metabolic pathways were co-enriched in the transcriptome and metabolome, including the plant signal transduction pathway, phenylpropane biosynthesis pathway, amino sugar and nucleotide sugar metabolism pathway, amino acid biosynthesis pathway, and carbon metabolism pathway. In the salt-tolerant tomato variety (IL7-5-5), 59 metabolic pathways were co-enriched in the transcriptome and metabolome, including the plant signal transduction pathway, amino acid biosynthesis pathway, carbon metabolism pathway, starch and sucrose metabolism pathway, and amino sugar and nucleotide sugar metabolism pathway. Further analysis showed that many differentially expressed genes and differential metabolites belonging to the plant hormone signal transduction and amino acid biosynthesis pathways were enriched in both varieties, indicating that the GABA-enhanced salt tolerance in tomato seedlings mainly occurred by regulating these two pathways.

## Figures and Tables

**Table 1:** Tukey HSD test results of MDA, H_2_O_2_, and O_2_^−^.

	LCK					LN					LNA					MCK					MN					MNA				
	LN	LNA	MCK	MN	MNA	LCK	LNA	MCK	MN	MNA	LCK	LN	MCK	MN	MNA	LCK	LN	LNA	MN	MNA	LCK	LN	LNA	MCK	MNA	LCK	LN	LNA	MCK	**MN**
MDA-Sig.	0.000	0.999	0.055	0.101	0.906	0.000	0.000	0.000	0.001	0.000	0.999	0.000	0.030	0.179	0.984	0.055	0.000	0.030	0.000	0.010	0.101	0.001	0.179	0.000	0.436	0.906	0.000	0.984	0.010	0.436
H_2_O_2_-Sig.	0.006	0.000	0.002	0.000	0.000	0.006	0.000	0.985	0.000	0.000	0.000	0.000	0.000	0.000	0.356	0.002	0.985	0.000	0.000	0.000	0.000	0.000	0.000	0.000	0.000	0.000	0.000	0.356	0.000	0.000
O_2_^−^-Sig.	0.000	0.000	0.250	0.000	0.000	0.000	0.000	0.000	0.000	0.046	0.000	0.000	0.000	0.665	0.000	0.250	0.000	0.000	0.000	0.000	0.000	0.000	0.665	0.000	0.000	0.000	0.046	0.000	0.000	0.000

**Table 2:** Tukey HSD test results of the antioxidant oxidase activity.

	LCK					LN					LNA					MCK					MN					MNA				
	LN	LNA	MCK	MN	MNA	LCK	LNA	MCK	MN	MNA	LCK	LN	MCK	MN	MNA	LCK	LN	LNA	MN	MNA	LCK	LN	LNA	MCK	MNA	LCK	LN	LNA	MCK	**MN**
SOD-Sig.	0.000	0.000	0.222	0.284	0.000	0.000	0.646	0.001	0.001	0.989	0.000	0.646	0.008	0.006	0.329	0.222	0.001	0.008	1.000	0.000	0.284	0.001	0.006	1.000	0.000	0.000	0.989	0.329	0.000	0.000
POD-Sig.	0.000	0.000	0.000	0.021	0.000	0.000	0.803	1.000	0.000	0.000	0.000	0.803	0.646	0.000	0.000	0.000	1.000	0.646	0.001	0.000	0.021	0.000	0.000	0.001	0.000	0.000	0.000	0.000	0.000	0.000
CAT-Sig.	1.000	0.010	0.122	0.581	0.009	1.000	0.010	0.128	0.598	0.010	0.010	0.010	0.659	0.151	1.000	0.122	0.128	0.659	0.847	0.646	0.581	0.598	0.151	0.847	0.146	0.009	0.010	1.000	0.646	0.146
APX-Sig.	0.966	0.000	0.471	0.604	0.000	0.966	0.000	0.162	0.231	0.000	0.000	0.000	0.001	0.001	0.930	0.471	0.162	0.001	1.000	0.004	0.604	0.231	0.001	1.000	0.003	0.000	0.000	0.930	0.004	0.003
GR-Sig.	0.033	0.064	0.000	0.000	0.326	0.033	0.000	0.003	0.000	0.698	0.064	0.000	0.000	0.008	0.002	0.000	0.003	0.000	0.000	0.000	0.000	0.000	0.008	0.000	0.000	0.326	0.698	0.002	0.000	0.000

**Table 3:** A summary of the number of differentially accumulated metabolites among samples.

Group Name	Diff_num	Up_num	Down_num
MCK vs. MN	275	191	84
MCK vs. MNA	267	193	74
MN vs. MNA	199	103	96
LCK vs. LN	263	146	117
LCK vs. LNA	295	184	111
LN vs. LNA	197	113	84

Note: Diff_num: the number of differential metabolites; Up_num: the number of up-regulated metabolites; and Down_num: the number of down-regulation metabolites.

**Table 4:** Sample output statistics.

Sample	ReadSum	BaseSum	GC (%)	N (%)	Q20 (%)	Q30 (%)
MCK-1	210,989,01	631,661,932,3	43.27	0.01	99.58	98.05
MCK-2	224,898,20	672,725,226,0	43.31	0	99.77	98.76
MCK-3	201,647,38	603,597,553,9	43.12	0	99.74	98.61
MN-1	241,837,54	723,080,861,3	42.61	0	99.76	98.68
MN-2	211,970,17	634,641,468,7	42.64	0.01	99.57	98.04
MN-3	190,959,64	571,555,020,3	42.44	0	99.68	98.26
MNA-1	211,511,26	633,403,635,7	42.53	0.01	99.58	98.04
MNA-2	208,902,37	625,623,122,6	42.43	0.01	99.59	98.02
MNA-3	217,020,72	649,555,330,2	42.6	0.01	99.59	98.17
LCK-1	220,143,47	658,988,952,0	43.13	0.01	99.41	97.44
LCK-2	190,725,61	570,922,363,9	43.44	0	99.71	98.42
LCK-3	245,052,22	733,943,717,4	43.08	0.01	99.42	97.21
LN-1	236,300,35	707,530,837,5	42.53	0	99.46	97.35
LN-2	198,370,73	593,281,792,2	42.6	0	99.74	98.59
LN-3	207,186,43	620,155,599,4	42.48	0.01	99.42	97.14
LNA-1	280,051,52	838,282,879,7	42.62	0.01	99.17	96.19
LNA-2	201,280,71	602,393,950,2	42.51	0	99.73	98.54
LNA-3	218,912,02	655,089,226,0	42.52	0	99.77	98.75

Note: Sample: sample name; ReadSum: total number of pair-end reads in the cleaned data; BaseSum: total number of bases in the cleaned data; GC (%): GC content in the cleaned data; N (%): percentage of base positions containing N in the cleaned data; Q20 (%): percentage greater than or equal to 20; and Q30 (%): percentage greater than or equal to 30.

**Table 5:** Comparison statistics.

Sample	Total Reads	Mapped Reads	Uniq Mapped Reads	Reads Map to ‘+’	Reads Map to ‘−‘
MCK-1	42,197,802	41,274,901 (97.81%)	40,430,291 (95.81%)	21,156,358 (50.14%)	21,210,461 (50.26%)
MCK-2	44,979,640	44,178,672 (98.22%)	43,231,943 (96.11%)	22,678,456 (50.42%)	22,723,535 (50.52%)
MCK-3	40,329,476	39,541,999 (98.05%)	38,751,243 (96.09%)	20,245,685 (50.20%)	20,284,739 (50.30%)
MN-1	48,367,508	47,537,504 (98.28%)	46,703,086 (96.56%)	24,263,826 (50.17%)	24,284,427 (50.21%)
MN-2	42,394,034	41,446,951 (97.77%)	40,632,075 (95.84%)	21,221,383 (50.06%)	21,252,385 (50.13%)
MN-3	38,191,928	37,495,686 (98.18%)	36,866,128 (96.53%)	19,111,655 (50.04%)	19,127,135 (50.08%)
MNA-1	42,302,252	41,430,976 (97.94%)	40,730,803 (96.29%)	21,112,541 (49.91%)	21,136,472 (49.97%)
MNA-2	41,780,474	40,916,853 (97.93%)	40,211,935 (96.25%)	20,857,926 (49.92%)	20,872,340 (49.96%)
MNA-3	43,404,144	42,433,987 (97.76%)	41,688,323 (96.05%)	21,637,750 (49.85%)	21,661,415 (49.91%)
LCK-1	44,028,694	42,788,530 (97.18%)	41,932,722 (95.24%)	21,898,669 (49.74%)	21,967,678 (49.89%)
LCK-2	38,145,122	36,888,310 (96.71%)	35,699,546 (93.59%)	19,269,038 (50.52%)	19,339,712 (50.70%)
LCK-3	49,010,444	47,831,969 (97.60%)	46,876,632 (95.65%)	24,474,276 (49.94%)	24,541,999 (50.08%)
LN-1	47,260,070	46,154,514 (97.66%)	45,342,161 (95.94%)	23,554,100 (49.84%)	23,580,854 (49.90%)
LN-2	39,674,146	38,876,335 (97.99%)	38,202,195 (96.29%)	19,841,724 (50.01%)	19,851,391 (50.04%)
LN-3	41,437,286	40,585,894 (97.95%)	39,921,706 (96.34%)	20,668,325 (49.88%)	20,688,261 (49.93%)
LNA-1	56,010,304	54,432,815 (97.18%)	53,433,695 (95.40%)	27,785,384 (49.61%)	27,838,453 (49.70%)
LNA-2	40,256,142	39,469,075 (98.04%)	38,750,951 (96.26%)	20,143,686 (50.04%)	20,155,587 (50.07%)
LNA-3	43,782,404	42,857,330 (97.89%)	42,103,081 (96.16%)	21,866,305 (49.94%)	21,879,328 (49.97%)

Note: Total Reads: number of clean sequenced reads; Mapped Reads: number of reads mapped to the reference genome and their percentage relative to the total number of reads; Uniq Mapped Reads: number of reads aligned to unique locations on the reference genome and percentage of napped reads; Reads Map to ‘+’: number of reads with chain of the reference genome and the percentage of reads; and Reads Map to ‘−’: number of reads with negative chains in the reference genome and the percentage of reads.

**Table 6:** Top 10 KEGG pathways in DEG/DEM for MN–MNA.

Pathway Definition	Number of DEGs	Number of DEMs
Plant hormone signal transduction	45	1
Biosynthesis of amino acids	26	2
Carbon metabolism	25	1
Starch and sucrose metabolism	21	1
Amino sugar and nucleotide sugar metabolism	19	1
Glutathione metabolism	16	3
ABC transporters	13	6
Cysteine and methionine metabolism	11	3
Alpha-linolenic acid metabolism	10	3
Fatty acid metabolism	10	1

**Table 7:** Top 10 KEGG pathways in DEG/DEM for LN–LNA.

Pathway Definition	Number of DEGs	Number of DEMs
Plant hormone signal transduction	29	1
Phenylpropanoid biosynthesis	17	2
Amino sugar and nucleotide sugar metabolism	17	1
Biosynthesis of amino acids	16	10
Carbon metabolism	16	3
Glutathione metabolism	13	2
Cyanoamino acid metabolism	12	8
Pentose and glucuronate interconversions	12	2
Fatty acid metabolism	12	1
Inositol phosphate metabolism	10	1

## Data Availability

All data are included in the article. RNA-seq raw data are available at the NCBI database (PRJNA1246248).

## Supplementary Materials

The following supporting information can be downloaded at https://www.mdpi.com/article/10.3390/ijms26115145/s1.
