# Supplementary material for: Molecular Mechanism of Exogenous GABA in Regulating Salt Tolerance in Tomato (Solanum lycopersicum L.)
Source: Int J Mol Sci. 2025 May 27;26(11):5145. doi: 10.3390/ijms26115145 (PMC12154988; doi:10.3390/ijms26115145)
Supplement: Supplementary file 1 [file ijms-26-05145-s001.zip › ijms-3600106-supplementary.pdf]

Table S1. Primer information.

Num	Gene	Forward sequence (5' to 3')	Reverse sequence (3' to5')
actin	actin-F	ATGCCATTCTCCGTCTTG	CGAGTTCCTGTTCATAGTCA
1	LOC101256090	GACTGAGAACCACCTTGT	CCAATACGAACACCTCCT
2	GS	ACTCCAGCCAAATGTAAACT	CCTTGTAGTGAGCATCTGATAT
3	LOC101254617	GGTTGTTGGTTGGGATTTC	TGTTCTCGCATTCCTCTC
4	NAOD	GGAACTTGTGGTAACTGGAA	GGTGGATGGTGCTTCAAA
5	LOC101245299	CAAGGTAACTACAACGATTGG	TGGCTCTTCACATCTTCAG
6	SAM1	CTGGTCTCACTGGTCGTA	CAGCCTGCCTTACAATGT
7	LOC100191111	CTGGTGCTGTGAAGATGT	CAGTTACCTGGTGGATCATAA
8	LOC101055547	ATCCAATCAAGCCACCTG	GCAGCAGTAGTCTTCTCAC
9	LOC101055583	TCACAAACACCACCATCAC	ATGACCTACCAATACTGGAAG A
10	GAI	GAGCAAGAAGCGAATCATAAC	CGGAGGTTGAGGAAGAATC
11	LOC101268226	AGGAGGTGATGATGAGGAT	TCGTTCAGACTGGTTGTG
12	PP2C-1	CTAACCCTCTGCCTGAAAG	CCACCACAATCACGGATAT

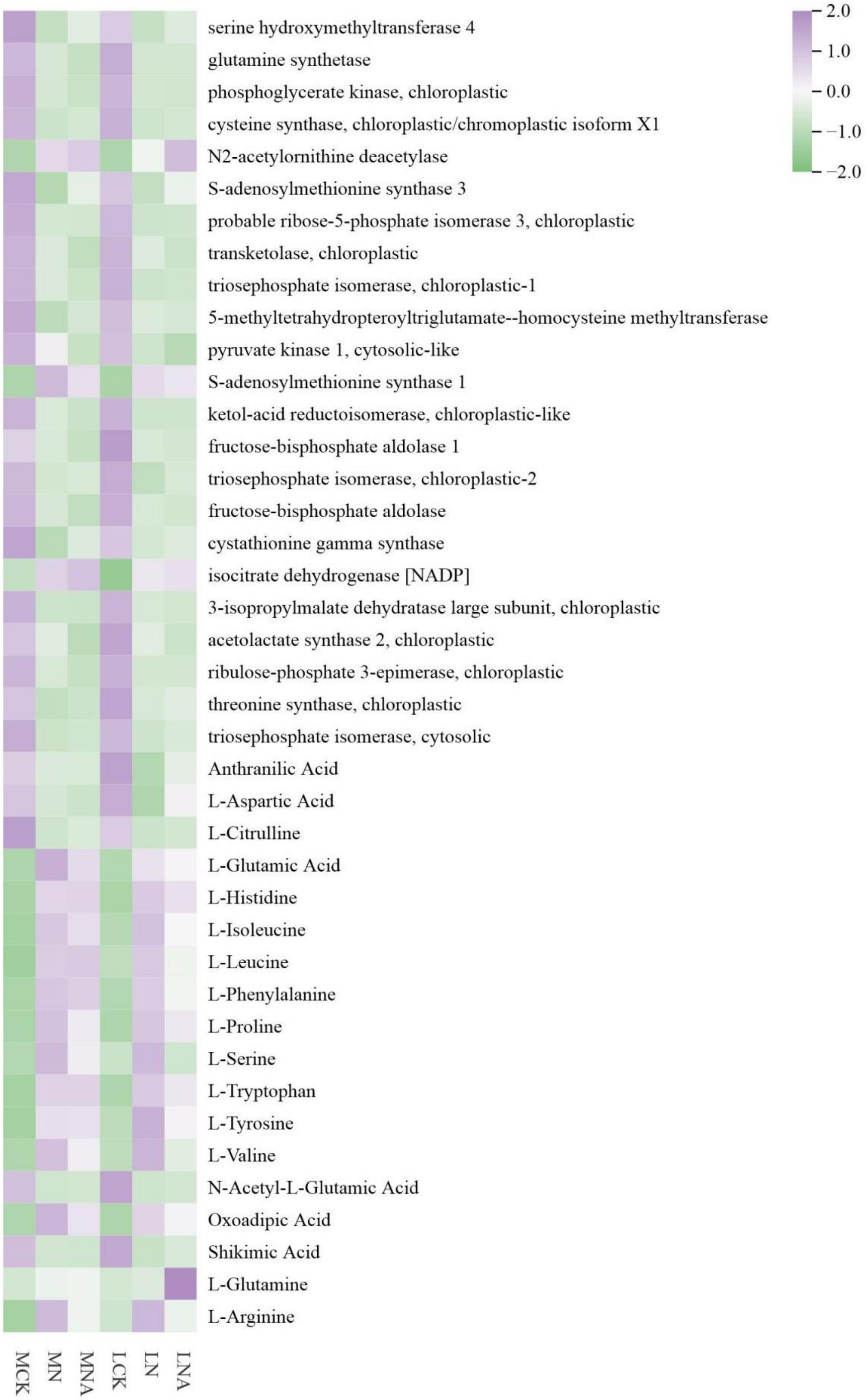

Figure S1. Heatmap of Differentially Expressed Genes and Metabolites in the Amino Acid Metabolism Pathway

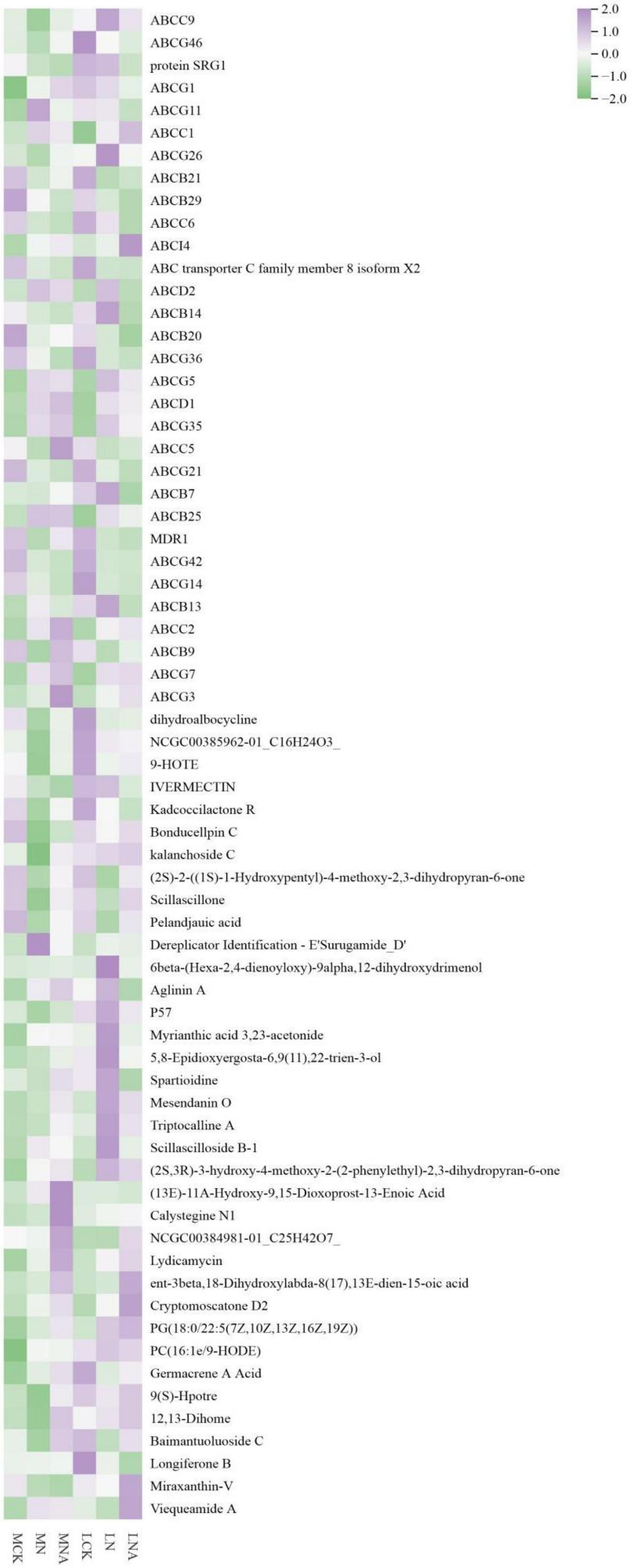

Figure S2. Heatmap of Differentially Expressed Genes and Metabolites in the ABC transporters Pathway

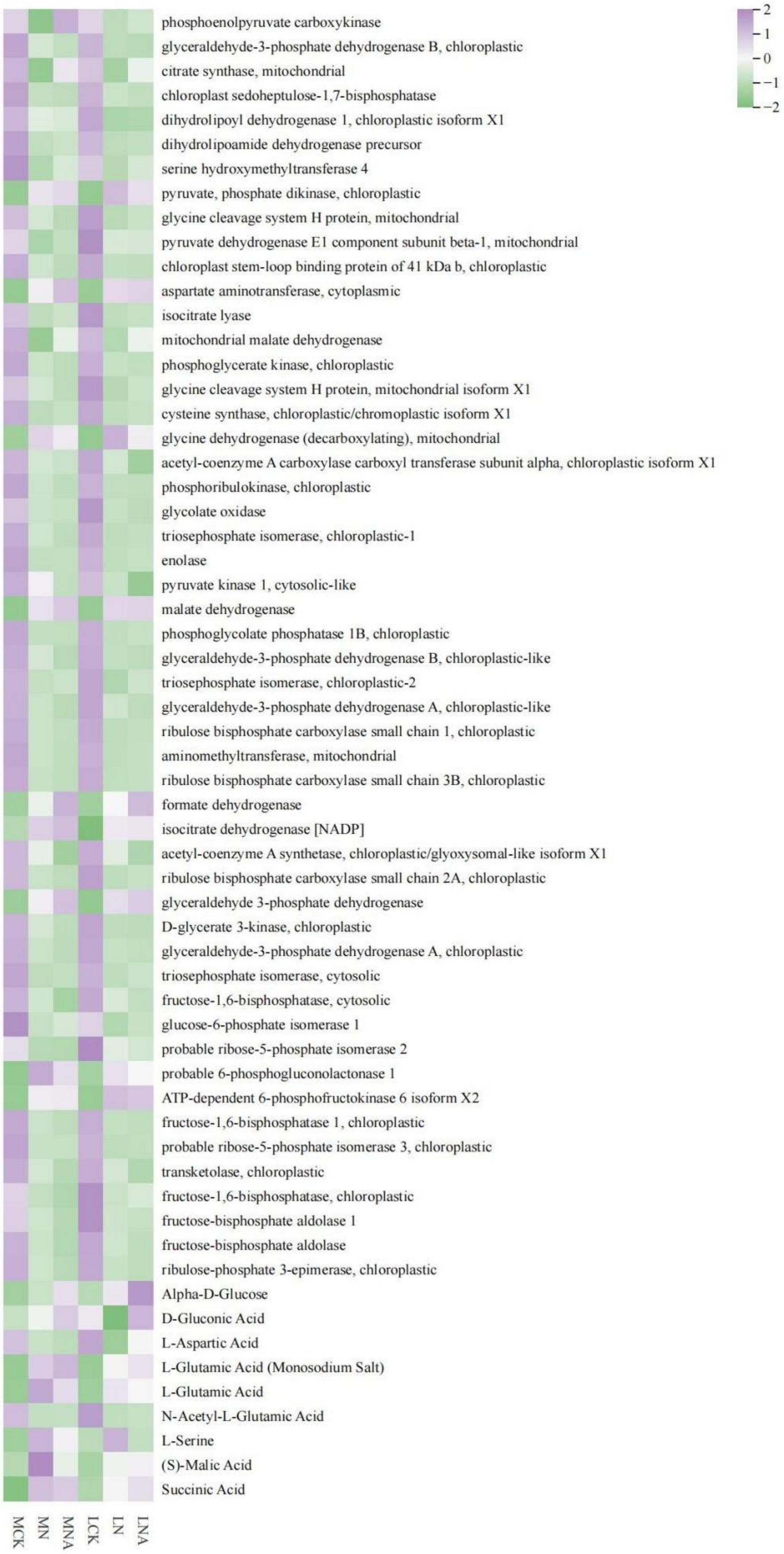

Figure S3. Heatmap of Differentially Expressed Genes and Metabolites in the Carbon metabolism Pathway
